# Potential Targets Other Than PSMA for Prostate Cancer Theranostics: A Systematic Review

**DOI:** 10.3390/jcm10214909

**Published:** 2021-10-24

**Authors:** Mathieu Gauthé, Paul Sargos, Eric Barret, Gaëlle Fromont-Hankard, Jean-Baptiste Beauval, Laurent Brureau, Gilles Créhange, Raphaële Renard-Penna, Charles Dariane, Gaëlle Fiard, Romain Mathieu, Guilhem Roubaud, Alain Ruffion, Morgan Rouprêt, Guillaume Ploussard, on behalf of the CC-AFU

**Affiliations:** 1 Department of Nuclear Medicine, Scintep, 38000 Grenoble, France; 2 Department of Radiotherapy, Institut Bergonié, 33000 Bordeaux, France; P.Sargos@bordeaux.unicancer.fr; 3 Department of Urology, Institut Mutualiste Montsouris, 75014 Paris, France; eric.barret@imm.fr; 4 Department of Pathology, CHRU, 37000 Tours, France; gaelle.fromont-hankard@univ-tours.fr; 5 Department of Urology, La Croix du Sud Hospital, 31445 Quint Fonsegrives, France; jbbeauval@gmail.com (J.-B.B.); g.ploussard@gmail.com (G.P.); 6 Department of Urology, CHU de Pointe-à-Pitre, University of Antilles, Inserm, EHESP, Irset (Institut de Recherche en Santé, Environnement et Travail)-UMR_S 1085, 97110 Pointe-à-Pitre, France; laurent.brureau@chu-guadeloupe.fr; 7 Department of Radiation Oncology Curie Institute, 75005 Paris, France; gilles.crehange@curie.fr; 8 Radiology, Pitie-Salpetriere Hospital, Sorbonne University, AP-HP, 75013 Paris, France; raphaelle.renard-penna@aphp.fr; 9 Department of Urology, Hôpital européen Georges-Pompidou, APHP, Paris–Paris University–U1151 Inserm-INEM, Necker, 75015 Paris, France; Charles.dariane@aphp.fr; 10 Department of Urology, Grenoble Alpes University Hospital, Université Grenoble Alpes, CNRS, Grenoble INP, TIMC-IMAG, 38000 Grenoble, France; gfromont@chu-grenoble.fr; 11 Department of Urology, CHU Rennes, 35000 Rennes, France; romain.mathieu@chu-rennes.fr; 12 Department of Medical Oncology, Institut Bergonié, 33000 Bordeaux, France; g.roubaud@bordeaux.unicancer.fr; 13 Service d’urologie Centre Hospitalier Lyon Sud, Hospices Civils de Lyon, Equipe 2-Centre d’Innovation en cancérologie de Lyon (EA 3738 CICLY)-Faculté de médecine Lyon Sud-Université Lyon 1, 69000 Lyon, France; alain.ruffion@chu-lyon.fr; 14 GRC 5 Predictive Onco-Uro, Urology, Pitie-Salpetriere Hospital, Sorbonne University, AP-HP, 75013 Paris, France; morgan.roupret@aphp.fr; 15 Institut Universitaire du Cancer Oncopole, 31000 Toulouse, France

**Keywords:** nuclear medicine, therapeutics, molecular imaging

## Abstract

Background: Prostate-specific membrane antigen (PSMA) is not sufficiently overexpressed in a small proportion of prostate cancer (PCa) patients, who require other strategies for imaging and/or treatment. We reviewed potential targets other than PSMA for PCa theranostics in nuclear medicine that have already been tested in humans. Methods: We performed a systematic web search in the PubMed and Cochrane databases, with no time restrictions by pooling terms (“prostate cancer”, “prostatic neoplasms”) and (“radioligand”, “radiotracer”). Included articles were clinical studies. The results were synthetized by the target type. Results: We included 38 studies on six different targets: gastrin-releasing peptide receptors (GRPRs) (*n* = 23), androgen receptor (*n* = 11), somatostatin receptors (*n* = 6), urokinase plasminogen activator surface receptor (*n* = 4), fibroblast activation protein (*n* = 2 studies) and integrin receptors (*n* = 1). GRPRs, the most studied target, has a lower expression in high-grade PCa, CRPC and bone metastases. Its use might be of higher interest in treating earlier stages of PCa or low-grade PCa. Radiolabeled fibroblast activation protein inhibitors were the most recent and promising molecules, but specific studies reporting their interest in PCa are needed. Conclusion: Theranostics in nuclear medicine will continue to develop in the future, especially for PCa patients. Targets other than PSMA exist and deserve to be promoted.

## 1. Introduction

Prostate Cancer (PCa) is the most prevalent cancer in men worldwide, accounting for 21% of all diagnosed cancers, and being the 2nd cause of death by cancer in men [1]. Current recommenfirst-lineline active treatments for nonmetastatic PCa is radical prostatectomy or definitive radiation therapy completed with androgen-deprivation therapy (ADT) in intermediate and high-risk disease; a deferred treatment being offered only in carefully selected patients [2,3]. In case of loco-regional recurrence after surgery, salvage radiation therapy plus ADT is offered to patients [4]. At later stages, ADT becomes the cornerstone of treatment in metastatic disease (at diagnosis or at recurrence), in combination with a novel hormonal agent therapy (NAD) (abiraterone, apalutamide, enzalutamide) or docetaxel chemotherapy [5,6]. Such long-term therapies mainly target the androgen receptor, are responsible for castration-related side effects that may significantly impact patients’ quality of life and inevitably lead to tumor resistance by cell adaptative pathways.

PCa is therefore a malignancy of interest to develop theranostic strategies. Theranostics, a term of increasing use in nuclear medicine, consists of the detection by imaging of the presence of a tumor cell characteristic by a ligand radiolabeled with a radionuclide suitable for imaging, allowing the implementation of targeted treatment with a ligand of this tumor cell characteristic. In recent years, theranostics in PCa has mainly been represented by prostate-specific membrane antigen (PSMA) ligands radiolabeled with gallium-68 or fluorine-18 for PET imaging and with lutetium-177 or actinium-225 for radioligand therapy (RLT). To date, these types of imaging and treatment are offered to metastatic castration-resistant prostate cancer (mCRPC) patients, after the failure of NAD therapy and at least 1 line of taxane chemotherapy [2,3]. A systematic review and meta-analysis reported an overall decline in serum prostate-specific antigen (PSA) of more than 50% (compared to its baseline value) in 43% of mCRPC patients who benefited from third-line [^177^Lu]Lu-PSMA RLT. Patients also presented fewer adverse effects than those treated with third-line NAD therapy or chemotherapy [7]. Moreover, the recent publication of the large worldwide phase III prospective trial “VISION”, reported that third-line RLT by [^177^Lu]Lu-PSMA-617 increased mCRPC patient imaging-based progression-free survival and overall survival by 5.3 and 4 months compared to standard care alone, respectively [8]. [^177^Lu]Lu-PSMA-based RLT efficacy in hormone-sensitive metastatic PCa is currently being investigated in other phase III trials. Furthermore, as it was demonstrated that PSMA overexpression in PCa was correlated with a higher Gleason score and lower androgen receptor expression, and that it was not sufficiently overexpressed in 5–10% of PCa patients [9,10], there is a need for the identification of other targets.

The purpose of this systematic review was to investigate targets other than PSMA for PCa theranostics in nuclear medicine that have already been injected into humans.

## 2. Materials and Methods

We performed a systematic web search in the PubMed and Cochrane databases, according to the Preferred Reporting Items for Systematic Reviews and Meta-analyses (PRISMA) guidelines, with no time restrictions until July 2021. We pooled the terms (“prostate cancer”, “prostatic neoplasms”) and (“radioligand”, “radiotracer”) using the Boolean operator AND. We also performed a manual search by consulting the references of the included web-searched articles. Data extraction was carried out by an author (M.G.) who is an expert in the field of nuclear medicine and PCa. The included published articles were all clinical studies reporting the use of radiolabeled ligands for imaging and therapy of PCa patients other than radiolabeled PSMA ligands. Preclinical studies, case reports and abstracts were not considered. Given the heterogeneity in terms of disease characteristics, clinical context, the low number of patients and the absence of randomized controlled trials, no meta-analysis was performed.

## 3. Results

### 3.1. Study Selection

**Figure 1:**
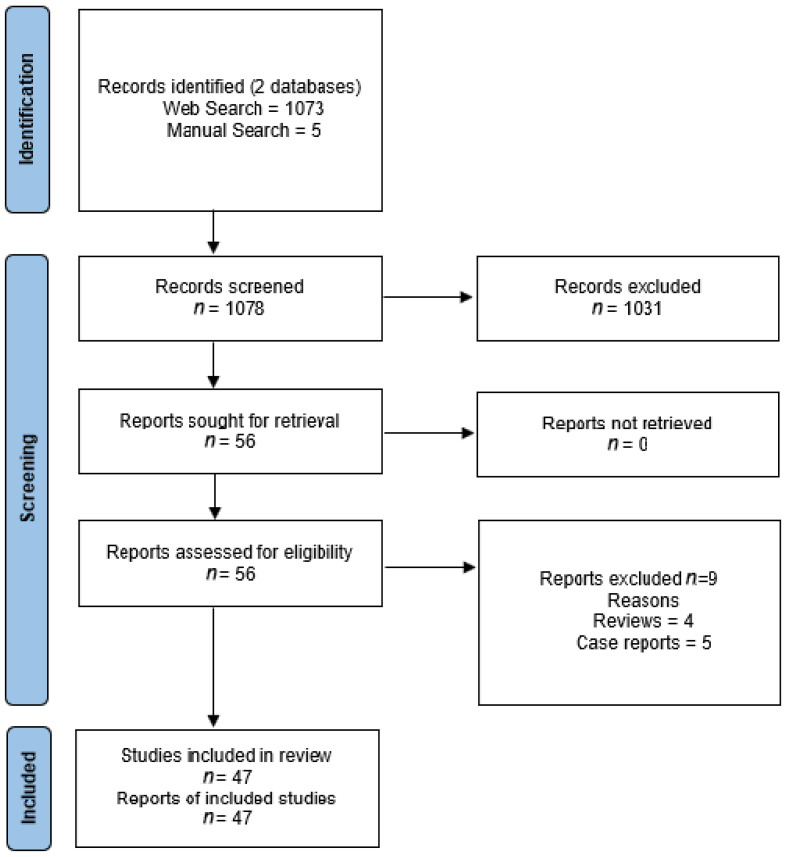
Preferred reporting items of systematic reviews and meta-analyses (PRISMA) flow diagram of selected studies.

The selection flow diagram adapted from the PRISMA recommendations is illustrated in Figure 1. We included 47 studies that investigated the use of radioligands that target six different receptors of tumor cells for imaging or therapy of PCa patients. The most studied receptors in PCa were gastrin-releasing peptide receptors (*n* = 23 studies) [11,12,13,14,15,16,17,18,19,20,21,22,23,24,25,26,27,28,29,30,31,32,33] and the androgen receptor (*n* = 11 studies) [34,35,36,37,38,39,40,41,42,43,44], followed by somatostatin receptors (*n* = 6 studies) [45,46,47,48,49,50], the urokinase plasminogen activator surface receptor (*n* = 4 studies) [51,52,53,54], the fibroblast activation protein (*n* = 2 studies) [55,56] and integrin receptors (*n* = 1 study) [57]. As only phase I and II studies were available, the risk of bias and uncertainty were not assessed.

### 3.2. Current Targets Other Than PSMA for Prostate Cancer Theranostics

#### 3.2.1. Gastrin-Releasing Peptide Receptors

Gastrin-releasing peptide receptors (GRPRs) are transmembrane G-protein coupled receptors whose overexpression in PCa has been known for a long time [58]. Radiolabeled ligands of GRPRs are mainly derived from a homolog of gastrin-releasing peptide (GRP), bombesin, a 14 amino acid initially isolated from the skin of the European fire-bellied toad [59]. Physiologically, GRP is implicated in multiple functions of the gastrointestinal tract and central nervous system [60]. It has been demonstrated in vitro that GRP increases PCa cell growth and invasion [61]. Most of the developed peptides are bombesin-like GRPR antagonists, as it was suggested that they had a better affinity to GRPRs than GRPR agonists, while they were not internalized in the cells [62]. Imaging PCa patients with radiolabeled bombesin-like peptides started in the early 2000s. The first report, a feasibility study using a bombesin-like peptide radiolabeled with technetium-99m for scintigraphy of 10 patients, including 4 metastatic PCa patients, was published in 2000 [11]. Three other reports concerning bombesin-like peptides radiolabeled with technetium-99m were published in 2002 (one PCa patient) [12], 2003 (8 PCa patients) [13] and 2014 (8 PCa patients) [14]. Since then, the potential interest of many bombesin-like peptides radiolabeled for PET imaging in multiple series of PCa patients and various clinical contexts has been reported several times (Table 1) [15,16,17,18,19,20,21,22,23,24,25,26,27,28,29,30]. In these studies concerning heterogeneous PCa patients, the reported per-patient positivity rates (at least one foci suggestive of PCa) ranged from 31 to 93%, which could be explained by the lower expression of GRPRs in high-grade PCa, CRPC and bone metastases [58,63]. The results of a study including a large homogenous series of patients are needed to draw further conclusions.

One of these bombesin-like peptides, RM2, was recently radiolabeled with lutetium-177 and used for RLT in 4 CRPC patients who previously demonstrated GRPR positivity on [^68^Ga]Ga-RM2 PET/CT [31]. Additional data are needed on more patients to draw conclusions on the efficacy of such treatment.

Finally, two first-in-human studies have recently reported the use of heterodimeric peptides radiolabeled with gallium-68 for PET imaging that targeted GRPR+integrin receptors [32] and GRPR+PSMA [33] in PCa patients. Here, again, more data are needed to assess the usefulness of such dual radioligands.

#### 3.2.2. Androgen Receptor

The androgen receptor is a nuclear receptor that is activated by steroid hormones, which is involved in sexual differentiation and bone and muscle growth and development [64]. The first clinical study that reported the use of a steroid-based radioligand, [^18^F]F-16β-fluoro-5α-dihydrotestosterone (FHDT), to the target androgen receptor in PCa patients was published in 2004 [34]. In this study that included 7 progressive mCRPC patients, FDHT PET was positive in 78% of the 59 metastatic lesions that were detected by FDG-PET (which was positive in 57/59 metastatic lesions) and “conventional imaging” (bone scan in all patients, CT scan in 4 patients and MRI in one patient) [34]. Overall, 11 studies reported heterogeneous results of FHDT PET in 323 patients (with possible duplicates) (Table 2), 256 of whom were mCRPC patients [34,35,36,37,38,39,40,41,42,43,44]. Few studies have reported the positivity rate of FDHT PET, and the standard of truth for detected lesions was questionable most of the time. The largest series that prospectively enrolled 133 mCRPC patients for both FHDT and FDG PET reported that patients who presented at least one lesion negative on FHDT PET and positive on FDG PET had the worst overall survival [40]. It was also reported that bone metastasis uptake on FDHT PET in mCRPC patients was correlated with survival, with patients presenting a higher uptake having a shorter survival [39]. In all cases, a large prospective study using a strong standard of truth for detected lesions is needed to validate the interest of FHDT PET. To date, no androgen radioligand radiolabeled with a radionuclide suitable for therapy has been tested in humans.

#### 3.2.3. Somatostatin Receptors

Somatostatin receptors (SSTRs) are a group of 5 subtype G protein-coupled transmembrane receptors that have a role in growth hormone, insulin and glucagon secretion, and in neuronal activity [65]. They are usually overexpressed in neuroendocrine tumors [65]. SSTRs have a low expression in the normal prostate, even if it increases with patient age and in hypertrophic and hyperplastic prostate [45]. Few studies have suggested that SSTRs expression could be increased in PCa, especially in mCRPC, and even without histological evidence of neuroendocrine differentiation [45,46,47,48,49,50]. Overexpression of SSTRs by PCa may be of interest, as targeted SSTRs therapies, including radioligands radiolabeled with radionuclides suitable for therapy approved for RLT of advanced neuroendocrine tumors, exist. In 1995, Nilsson et al. reported a lesion-based detection rate of 37% in 31 mCRPC patients (346 lesions) explored by somatostatin scintigraphy [46]. In this study, one patient with 20% positive lesions on somatostatin scintigraphy was treated with somatostatin analog, which led to an 80% decrease in lesion uptake, but had no effect on PSA serum levels [46]. Other studies using SSTRs radioligand radiolabeled with gallium-68 for PET imaging reported patient-based detection rates ranging from 48 to 65%, with lower lesion-based detection rates, and noted that radiotracer uptake was usually low [47,48,49]. However, it was suggested that patients presenting a high uptake of their PCa lesions by SSTRs PET could be considered for SSTR-targeted therapies other than RLT [48,49].

#### 3.2.4. Urokinase Plasminogen Activator Surface Receptor

The urokinase plasminogen activator surface receptor (uPAR) is a cell membrane glycoprotein that is implicated in the plasminogen activation system [51], whose overexpression was demonstrated in PCa and considered a biomarker for aggressive disease and poor prognosis [51,52]. Two studies reported the use of a radioligand of uPAR (AR105) radiolabeled with copper-64 and gallium-68 for PET imaging, which was first injected in 4 PCa patients in 2015 and 6 PCa patients in 2017 [51,52]. More recently, AR105 radiolabeled with gallium-68 PET/MRI was reported to be correlated with the Gleason score in 27 newly diagnosed PCa patients, and the authors suggested that this radiotracer might replace biopsy repetition in PCa active surveillance [53]. No use of an uPAR radioligand radiolabeled with a radionuclide suitable for therapy has been published yet, but a high baseline SUVmax on [^68^Ga]Ga-AR105 PET/CT was reported to be associated with worse overall survival and increased symptomatic skeletal events in 17 mCRPC patients treated with radium-223 therapy [54]. In this study, the authors emphasized that uPAR radioligand uptake was low in bone metastases and that bone lesion delineation needed another imaging modality and that its uptake at 2 cycles of radium-223 therapy was not associated with disease progression [54].

#### 3.2.5. Fibroblast Activation Protein

Fibroblast activation protein (FAP) is a type II transmembrane serine protease that is expressed on the cell surface of activated stromal fibroblast in 90% of epithelial tumors, wound healing tissues and fibrotic diseases [66]. FAP is suspected to have both antitumor and tumorogenic functions [67]. It could promote tumorigenesis of tumor cells by remodeling the extracellular matrix, increasing invasive capability, promoting monocyte chemoattractant protein 1 and suppressing T-cell functions [67]. Fibroblast activation protein inhibitors (FAPis) are quinoline-based inhibitors that have recently been radiolabeled with gallium-68 for PET imaging of various cancers, including CaP, in 2 studies [55,56]. FAP expression by PCa cells assessed by [^68^Ga]Ga-FAPI-04 PET uptake is reported to be sufficient in mCRPC patients to consider the use of FAPI radiolabeled with a radionuclide suitable for therapy in those patients [68]. Such radiotracers are still in development for clinical use.

#### 3.2.6. Integrin Receptors

Integrins are a group of transmembrane cell adhesion receptors that enable the attachment of the cell to the extracellular matrix and signal transduction [69]. αvβ3 integrin, which is implicated in tumor angiogenesis, was identified as suitable for targeted therapies in oncology, and its overexpression in PCa was reported [70]. In addition to the dual integrin-GRPR targeting PET radiotracer mentioned above [32], one short series reported the use of a radioligand radiolabeled with fluorine-18 for PET imaging that targets αvβ3 integrin, [^18^F]F-Galacto-RGD, in 12 PCa patients with known bone metastases [57]. In this study, the detection rate of bone metastases by [^18^F]F-Galacto-RGD PET was compared to that of bone scan which was considered the standard of truth for bone metastasis detection [57]. However, the authors indicated that [^18^F]F-Galacto-RGD PET could be valuable for patient screening before αvβ3-targeted therapies [57].

## 4. Discussion

### 4.1. Current Targets Other Than PSMA for Prostate Cancer Theranostics

While radiolabeled PSMA ligands are of increasing use worldwide, only a few potential targets other than PSMA for PCa theranostics in nuclear medicine have already been tested in humans, and only 10 recruiting clinical trials are currently reported on ClinicalTrial.gov (Table 3).

GRPRs are the most developed and studied targets. To date, only GRPRs have a radioligand available for both imaging and therapy that has already been tested in CRPC patients [31]. Unfortunately, GRPRs have lower expression in high-grade PCa, CRPC and bone metastases [15,63], and their use in such patients may be less effective. However, GRPRs radioligands might be of higher interest in treating earlier stages of PCa or low-grade PCa. Five recruiting clinical trials are currently exploring the usefulness of GRPR radioligands in PCa patients before initial definitive therapy (Table 1).

Radiolabeled FAPis are the most recent and promising molecules that have been developed and tested in multiple tumor types. They are believed to be sufficiently overexpressed in CRPC to perform RLT [68]. RLT with FAPis have recently been tested in humans but not in PCa [71]. Further specific studies reporting radiolabeled FAPis interest in PCa for diagnosis or therapy are needed.

Targeting the androgen receptor was an interesting option, but it appears that the overexpression of such receptors is heterogeneous, providing disappointing results.

SSTRs could have been targets of interest in neuroendocrine differentiation of PCa since it exists a theranostic pair that binds these receptors. Unfortunately, it was also suggested that SSTRs overexpression in that specific population was heterogeneous and not sufficient to perform RLT [50].

To date, very few studies have reported the use of molecular imaging radioligands of uPAR receptor and integrin receptors in a small number of PCa patients, and more data are needed about these potential targets.

### 4.2. Future Directions for Prostate Cancer Theranostics in Nuclear Medicine

The ideal target for a theranostic application should be sufficiently and exclusively overexpressed by PCa cells and have a ligand that could be radiolabeled with both radionuclides for imaging and therapy. Moreover, high receptor/ligand binding affinity and stability, and when appropriate a sufficient ligand–receptor cell internalization duration, would optimize the cytotoxic effect of RLT applications on PCa cells. Currently, radiolabeled PSMA ligands meet almost all of these criteria and are becoming the reference standard for theranostic in PCa. In view of the current scientific literature, the most promising targets to study in the future seem to be FAP for metastatic PCa and CRCP, and GRPRs for localized disease and low-grade PCa. Furthermore, chimeric antigen receptor T cell immunotherapy targeting PSMA has recently been tested preclinically [72]. Such treatments might also become an interesting investigative direction in the close future, paving the way for innovative therapeutic strategies in advanced PCa, alone or in combination with life-prolonging agents.

### 4.3. Limitations

This review has limitations mainly due to its very specific subject. We limited the included articles to those that reported results in humans. We chose to do so because many molecules do not go beyond preclinical assessment and will not necessarily be of interest in a clinical setting. The second main limitation is that we did not consider congress abstracts. Here, again, this was because a significant proportion of this type of communication will never be peer-reviewed and published in scientific journals. Finally, meta-analyses were not performed for potential targets because the reported data were limited and heterogeneous.

In conclusion, theranostics in nuclear medicine will continue to develop in the future. PCa patients, especially mCRPC patients, need new therapeutic approaches that offer hope for complementary lines of treatments when standard treatments fail. Other targets exist beyond PSMA for PCa theranostics that deserve to be promoted.

## Figures and Tables

**Table 1:** Studies reporting the use of radiolabeled bombesin-like peptides for prostate cancer PET imaging of gastrin-releasing peptide receptors.

Author Year	Peptide Radionuclide	*n*	Clinical Context	Positivity Rate	Se	Sp	Acc
Kähkonen et al. 2013 [15]	RM2 Gallium-68	14	Primary staging = 11 Recurrence = 3	48%	T = 89% N = 67%	T = 81%	T = 83%
Wieser et al. 2014 [16]	CB-TE2A-AR06 Copper-64	4	Primary staging = 4	75%	Ns	Ns	Ns
Sah et al. 2015 [17]	BAY 864367 Fluorine-18	10	Primary staging = 5 Recurrence = 5	50%	Ns	Ns	Ns
Maina et al. 2016 [18]	SB3 Gallium-68	9	Metastatic = 9	55%	Ns	Ns	Ns
Minamimoto et al. 2016 [19]	RM2 Gallium-68	7	Recurrence = 7	86%	Ns	Ns	Ns
Nock et al. 2017 [20]	NeoBOMB1 Gallium-68	4	Ns	Ns	Ns	Ns	Ns
Wieser et al. 2017 [21]	RM2 Gallium-68	16	Recurrence = 16	63%	Ns	Ns	Ns
Minamimoto et al. 2018 [22]	RM2 Gallium-68	32	Recurrence = 32	72%	Ns	Ns	Ns
Zhang et al. 2018 [23]	RM26 Gallium-68	28	Primary staging = 17 Recurrence = 11	82%	Ns	Ns	Ns
Gnesin et al. 2018 [24]	MJ9 Gallium-68	5	Recurrence = 5	Ns	Ns	Ns	Ns
Fassbender et al. 2019 [25]	RM2 Gallium-68	15	Primary staging = 15	93%	69%		63%
Touijer et al. 2019 [26]	RM2 Gallium-68	16	Primary staging = 16	100%	85%	67%	79%
Hoberück et al. 2019 [27]	RM2 Gallium-68	16	Primary staging = 2 Recurrence = 12 Other = 2	31%	Ns	Ns	Ns
Fassbender et al. 2020 [28]	RM2 Gallium-68	8	Primary staging = 8	Ns	Ns	Ns	Ns
Bakker et al. 2021 [29]	SB3 Gallium-68	10	Primary staging = 10	80%	88%	88%	Ns
Baratto et al. 2021 [30]	RM2 Gallium-68	50	Recurrence = 50	70%	Ns	Ns	Ns

Se: sensitivity; Sp: specificity; Acc: accuracy; Ns: not specified.

**Table 2:** Studies reporting the use of [^18^F]F-16β-fluoro-5α-dihydrotestosterone for prostate cancer PET imaging of androgen receptor.

Author	*n*	Population	Positivity Rate	
Year			Patient-Level	Lesion-Level
Larson et al. 2004 [34]	7	mCRPC	100%	97%
Zanzonico et al. 2004 [35]	7	Recurrence	Ns	Ns
Dehdashti et al. 2005 [36]	20	Advanced PCa (8 mCRPC)	63%	59%
Beattie et al. 2010 [37]	13	mCRPC	Ns	Ns
Fox et al. 2011 [38]	20	mCRPC	Ns	Ns
Vargas et al. 2014 [39]	38	mCRPC	74–95%	44–55%
Fox et al. 2018 [40]	133	mCRPC	Ns	87%
Vargas et al. 2018 [41]	27	mCRPC	Ns	Ns
Jansen et al. 2019 [42]	27	mCRPC	Ns	Ns
Kramer et al. 2019 [43]	14	mCRPC	Ns	Ns
Cysouw et al. 2019 [44]	14	mCRPC	Ns	Ns

PCa: prostate cancer; mCRPC: metastatic castration-resistant prostate cancer; Ns: not specified.

**Table 3:** Currently recruiting clinical studies available on ClinicalTrial.gov concerning the use of radiolabeled ligands of potential targets other than PSMA for prostate cancer theranostics (accessed 20 August 2021).

NCT Number	Category Type	Peptide Radionuclide	Phase	Population Number of Planned Patients	Endpoints
NCT04264208	GRPR Imaging	RM2 Gallium-68	2	PCa patients scheduled for HDR Brachytherapy *n* = 100	Definition of [^68^Ga]Ga-RM2 PET/MRI detection rate versus mpMRI Definition of [^68^Ga]Ga-RM2 PET/MRI ability to assess changes response to treatment Determination of progression-free survival
NCT03949517	GRPR Imaging	RM2 Gallium-68	1/2	PCa patients scheduled for HDR or HIFU *n* = 10	Evaluation of PCa response to HIFU or HDR Therapy
NCT03809078	GRPR Imaging	RM2 Gallium-68	2	Suspected PCa *n* = 20	Evaluation of [^68^Ga]Ga-RM2 PET/MRI for biopsy guidance in patients with suspected PCa
NCT03698370	GRPR Imaging	NeoBOMB1 Gallium-68	2	Recurrent PCa *n* = 50	To evaluate gallium [^68^Ga]Ga-NeoBOMB1 PET/MRI for detection of recurrent PCa after initial definitive therapy
NCT03606837	GRPR Imaging	RM2 Gallium-68	2	PCa patients scheduled for prostatectomy *n* = 15	Determination of uptake intensity assessed with median SUV
NCT03113617	GRPR Imaging	RM2 Gallium-68	2	PCa patients scheduled for prostatectomy *n* = 90	Determination of [^68^Ga]Ga-RM2 PET/CT detection rate in intermediate and high-risk PCa patients prior to prostatectomy.
NCT02624518	GRPR Imaging	RM2 Gallium-68	2/3	Recurrent PCa *n* = 125	Determination of [^68^Ga]Ga-RM2 PET/MRI detection rate in recurrent PCa after initial definitive therapy
NCT00588185	Androgen receptor Imaging	FHDT Fluorine-18	Ns	Progressive PCa *n* = 300	Determination of the accumulation and biodistribution of FDHT in patients with progressive PCa
NCT04457232	FAP Imaging	FAPi-46 Gallium-68	1	Metastatic recurrent PCa *n* = 30	Definition of the biodistribution of [^68^Ga]Ga-FAPi-46 in normal and cancer tissues of PCa patients
NCT04000776	SSTR Imaging	Octreotate Gallium-68	Ns	mCRPC patients *n* = 100	Determination of the prevalence of mCPRC intrapatient intermetastasis polyclonality and neuroendocrine using PET/CT triple tracer PSMA/FDG/OCTREOTATE imaging and their eligibility for radioligand therapy

GRPR: gastrin-releasing peptide receptor; FAP: fibroblast activation protein; SSTR: somatostatin receptor; PCa: prostate cancer; mCRPC: metastatic castration-resistant prostate cancer; HIFU: High intensity focused ultrasound; HDR: High dose-rate; Ns: not specified.

## Data Availability

Data sharing not applicable.
